# The Dose-Response Relationship between Alcohol Consumption and the Risk of Type 2 Diabetes among Asian Men: A Systematic Review and Meta-Analysis of Prospective Cohort Studies

**DOI:** 10.1155/2020/1032049

**Published:** 2020-08-24

**Authors:** Manman Han

**Affiliations:** Suzhou Hospital Affiliated To Anhui Medical University, No. 299 Bianhe Middle Road, Suzhou, AnHui 234000, China

## Abstract

The objective of this review was to provide a summary of the literature on the dose-response relationship between alcohol consumption and risk of type 2 diabetes (T2D) in Asian populations, particularly men. The present study was recorded in PROSPERO as CRD 42019121073. We searched the PubMed-Medline, Web of Science, and Cochrane Library for studies published in any language since the database inception to January 2019. Prospective cohort studies were included in the meta-analysis. Relative risks (RRs) and 95% confidence intervals (CIs) were calculated for random-effects models and dose-response meta-analyses. In total, 8 prospective cohort studies were included. High alcohol intake was significantly associated with increased risk of T2D (RR = 1.16, 95% CI: 1.04–1.29; Q statistic *p* = 0.326) compared to the lowest category of alcohol intake. Nonlinear association was observed between alcohol consumption and T2D risk in men (*p* = 0.003). Dose-wise, consuming ≤57 g/day of alcohol was not associated with the risk of T2D in this study; however, alcohol intake >57 g/day was associated with increased risk of T2D in men. Overall, the association between alcohol consumption and T2D among Asian men was J-shaped. Lifestyle recommendations for prevention of T2D should include advice on limiting alcohol intake. This trial is registered with Prospero registration: CRD 42019121073.

## 1. Introduction

Type 2 diabetes (T2D) and its complications pose a major threat to global health and present challenges to patients, health-care systems, and national economies [1]. According to the latest report in the International Diabetes Federation (IDF) Diabetes Atlas [2], the global prevalence of diabetes among adults was 9.1%, which is equivalent to 425 million adults with diabetes worldwide. More than 90% of these cases are T2D. The prevalence of T2D has dramatically increased in low- and middle-income countries, especially in Asia [2, 3]. On the number of cases of diabetes among Asian countries, China has ranked number one, with 114.4 million people of diabetes [2]. Additionally, diabetes and its complications tend to develop at a younger age among Asian populations than among Caucasian populations, leading to increased risk of premature diabetes-related deaths [4]. The reasons for the spike in T2D incidence are multiple, with genetic and environmental components suspected to play a major role. Nevertheless, while genetics might play a role in determining an individual's response to environmental changes, the root causes of the T2D epidemic among Asian populations are likely to be diet, lifestyle, and body mass index increase (BMI) [5]. In fact, previous studies have shown that lifestyle interventions, such as eating a healthy diet and exercising daily for at least 30 min, reduce the risk for T2D [6].

Reducing alcohol consumption might also mitigate the risk of developing T2D. Previously, a meta-analysis reported that moderate consumption of alcohol might reduce the risk of T2D, while high alcohol intake might increase the risk of T2D, following a U- or J-shaped relationship for new cases of T2D [7–9]. In addition, a recent meta-analysis showed moderate alcohol consumption was associated with a lower risk of T2D among women and non-Asian populations, whereas heavy alcohol consumption did not affect the risk of T2D in these groups [10, 11]. However, a meta-analysis of studies on Asian populations has not been reported to date. Historically, alcohol intake has been widespread among Asian men, but not among Asian women, and the studies examining the impact of alcohol on the risk of T2D in Asian populations have focused primarily on men [12–15]. Therefore, a meta-analysis was warranted to provide a summary of the existing literature and clarify the association between average daily alcohol consumption and T2D risk among Asian men.

## 2. Materials and Methods

This meta-analysis was performed according to the 2009 Preferred Reporting Items for Systematic Reviews and Meta-Analysis statement (PRISMA Checklist) [16]. The present study protocol was prospectively recorded in PROSPERO as CRD 42019121073.

### 2.1. Literature Search

PubMed, Web of Science, and the Cochrane Library databases were searched for studies that reported on the relationship between alcohol consumption and T2D, published in any language, since the start of the database coverage until January 2019. The search terms included alcohol-related terms (“alcohol”, “ethanol”, “drink∗,” “beer”, “wine”, “liquor” or “brandy”), plus diabetes-related terms (“diabet∗”, “type 2 diabetes”, “type 2 diabetes mellitus”, “T2D∗”, “non-insulin-dependent diabetes” or “NIDDM”), and plus a term indicative of a prospective study design (“prospective,” “cochort”, or “incidence”). We searched previously published relevant systematic reviews and meta-analysis to identify additional studies that might have been missed in database searches [7–11, 17]. In addition, we checked the references of included journal articles.

### 2.2. Study Selection

The inclusion criteria for studies were the following: (I) participants aged >18 years; (II) a prospective design; (III) exposure was alcohol consumption; (IV) outcome was incidence of type 2 diabetes; and (V) study provided the hazard ratio (HR) or relative risks (RRs) ratio with corresponding 95% confidence intervals (CIs) or data necessary to calculate them. We excluded reviews, editorials, studies involving non-Asian populations, and letters and editorials without sufficient data. Studies of other exposures and diseases were also excluded. Studies were excluded if reported consumption could not be converted into grams per day, and any abstention category included current drinkers. As the association between alcohol consumption and T2D risk has previously been reported as nonlinear, to obtain accurate estimates in this meta-analysis, studies could only be included if consumption was reported for multiple levels, including nondrinkers. As such, studies that did not report sufficient data were excluded. Finally, case-control studies were excluded due to the risk of bias associated with unreported confounding.

### 2.3. Data Extraction and Methodological Quality Assessment

Two reviewers independently identified eligible studies, extracted the relevant data, and performed the quality assessment of included studies. Authors were contacted for clarification in cases where essential information was not reported in full in published articles. Data inconsistencies were resolved by discussion, in consultation with an investigator not involved in the data extraction and conversion process. The final dataset was resolved by discussion, in consultation with an investigator not involved in the data extraction and conversion process. The final dataset was established by discussion and consensus. We performed a methodological quality assessment of the included studies using the Newcastle-Ottawa Scale (NOS) [18], which is a 9-star scale used to evaluate 3 items: selection (0–4stars), comparability (0–2stars), and outcome (for cohort study, 0–3 stars). We grouped the identified studies into 0–3, 4–6, and 7–9 stars categories, which corresponded to low-, medium-, and high-quality studies, respectively. The following information was subsequently extracted and tabulated: author (publication year), country (follow-up period), assessment of exposure, sample, participants' age at baseline, T2D cases (at end of follow-up), alcohol consumption categories, adjusted factors, and NOS score.

### 2.4. Statistical Analysis

We examined the relationship between alcohol intake and risk of T2D based on the effect estimates (risk ratios (RR) or hazard ratios (HR)) and the corresponding 95% confidence intervals (CI), determined for all studies. We extracted the maximally adjusted RRs or HRs and CIs. First, the overall relationship between alcohol consumption and T2D risk was estimated with a random-effects model, based on summary RRs and 95% CIs for different alcohol intake levels compared with none alcohol intake [19]. Next, we quantified the association between alcohol consumption and T2D risk as the weighted mean of the logarithm of RR estimates associated with the highest versus the lowest category (never drank) category, using fix or random effect models [20, 21]. Third, we estimated study-specific dose-response slopes by relating the logarithm of the RRs for different exposure levels to their corresponding alcohol content, using the method described by Greenland and coworkers [22, 23]. We converted all measurements into grams per day and defined one drink as 12 g of alcohol intake. Each category was assigned exposure value that corresponded to the mid-point of this category's range of alcohol intake. For the highest category, which was open-ended, we assumed the width of the interval to be the same as in the preceding category.

Random-effect models were used when there was evidence of heterogeneity. Heterogeneity between studies was investigated using the Q statistic [24], and we considered *p* values of <0.10 indicative of significant heterogeneity [25, 26]. Subgroup analyses were conducted for the T2D risk based on age, the number of participants/cases, the NOS score, country where the study was based, and follow-up duration. Finally, we performed sensitivity analyses by removing a study from the meta-analysis. Several methods were used to check for potential publication bias. According to Egger and colleagues, publication bias assessment is not reliable for estimates derived from fewer than 10 pooled studies [27]. Therefore, a funnel plot was used to evaluate any publication bias among the included studies [28].

## 3. Result

### 3.1. Studies and Patient Characteristics

**Figure 1:**
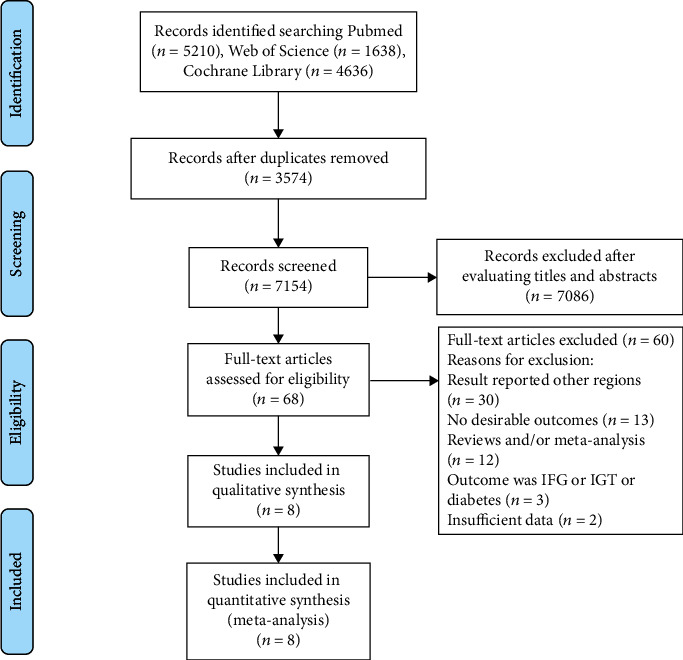
Flow diagram of the literature search and study selection process in the meta-analysis.

Details of study inclusion are presented in Figure 1. We identified 11,484 articles in our initial electronic search; a total of 11,413 were excluded because they were duplicate or irrelevant articles. Following initial screening, 71 potentially eligible studies were selected. After detailed evaluations, 8 prospective studies were included in the meta-analyses [12–15, 29–32]. The 8 included studies reported data on 89,842 individuals (3,975 T2D cases), enrolled in prospective cohort studies. The range of follow-up period was 4-12.2 years. A total of 4 studies originated in Japan [15, 29, 31, 32], 2 in Korea [13, 30], and 2 in China [12, 14]. The detail of the included studies are presented in Table 1. Quality assessment scores [18] for each included study are presented in Table 2.

### 3.2. Alcohol Consumption and T2D

**Figure 2:**
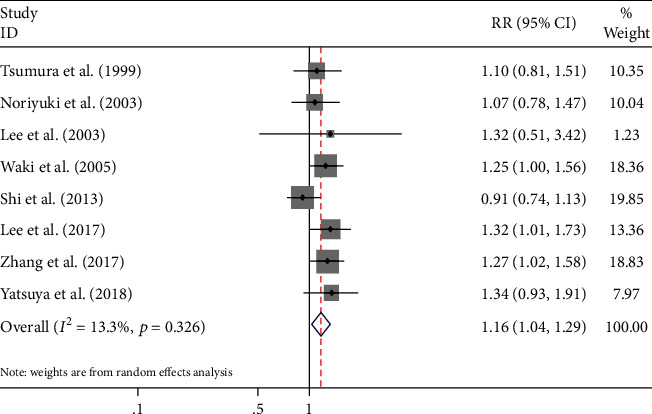
The forest plot of alcohol consumption and the risk of T2D in men. (highest vs. lowest).

All 8 studies included in the meta-analysis evaluated the association between alcohol intake and the T2D risk [12–15, 29–32]. The summary RRs showed that alcohol intake, compared with no alcohol intake, was not associated with risk of T2D among Asian men (RR = 1.01, 95% CI: 0.88–1.15, *p* = 0.931; Q statistic, *p* < 0.001; *I*^2^ = 76%) (Supplementary Figure 1). However, there was a significant association between high of alcohol consumption and T2D risk among Asian men (highest vs. lowest consumption category RR = 1.16, 95% CI: 1.04-1.29, *p* = 0.006; Q statistic, *p* = 0.326; *I*^2^ = 13.3%) (Figure 2).

### 3.3. Dose-Response Meta-Analysis

**Figure 3:**
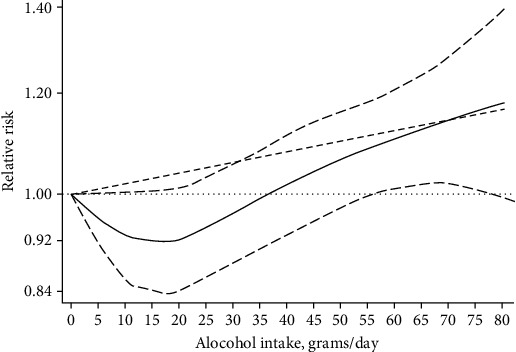
Dose-response relations between alcohol intake and relative risks of T2D in men.

Eight cohorts were included in the dose-response analysis, estimating the relationship between alcohol intake and risk of T2D [12–15, 29–32]. There was a nonlinear dose-response relationship between alcohol intake and T2D risk among Asian men (*p*_nonlinearity_ = 0.003) (Figure 3). Alcohol intake of 0-57 g/day was not associated with the risk of T2D among Asian men. However, alcohol intake of >57 g/day was associated with increased risk of T2D among Asian men.

### 3.4. Sensitivity Analysis and Subgroup Analysis

Sensitivity analysis showed that removing one study with high heterogeneity from the present study's analysis sample did not affect the overall effect estimates (Supplementary Figure 2). The dose-response relationship retained its nonlinear shape in sensitivity testing. In subgroup analyses, the highest dose of alcohol intake was associated with an increased risk of T2D among the men under 50 years of age, individuals based in Japan and Korea, as well as participants of studies conducted before 2010, studies that included <10,000 individuals and<500 cases, and studies where the NOS score was ≥8 (Supplementary Table 1).

### 3.5. Publication Bias

The funnel plots showed no evidence of publication bias for any subgroup (Supplementary Figure 3).

## 4. Discussion

To our knowledge, this is the first systematic review and meta-analysis of studies on the association between alcohol consumption and the risk of type 2 diabetes (T2D) among Asian men. Our results suggest that the deleterious effects of high intake on T2D risk are statistically significant in Asian men. Moreover, our findings suggest that low to moderate alcohol intake does not reduce the risk of T2D, whereas heavy alcohol intake is associated with increased risk of T2D; the relationship between alcohol consumption and the risk of T2D among Asian men followed a J-shape in this study.

In the last decades, several meta-analysis synthesizing findings on the relationship between alcohol consumption and T2D risk have become available; however, the results remain controversial. Some of these meta-analyses have shown a J-shaped relationship between outcome and exposure in population [9]. However, other studies reported that moderate alcohol consumption was protective against diabetes, while heavy alcohol consumption had no effect on the incidence of T2D, following a U-shape [11]. Moreover, while some studies reported that moderate alcohol consumption increased the risk of developing diabetes [33]. Knott et al. suggested that moderate drinking was beneficial for women and non-Asian populations [10]. For these contradictions, ethnicity, dietary differences, sample size, and individual choosing criteria may be the main explanation in these single studies. In the present study, the relationship between alcohol consumption and T2D risk among Asian men followed a J-shaped dose-response curve.

With respect to the dose-response between alcohol consumption and the risk of type 2 diabetes, we found that moderate alcohol exposure had no effect on the risk of T2D among Asian men. In contrast to previous meta-analyses, moderate alcohol consumption lowered the risk of diabetes [9, 10, 17]. These different results must be qualified by gender and ethnicity [34]. Studies have shown that there are racial/ethnic differences in drinking culture. For instance, Knott and colleagues suggested that moderate drinking was beneficial for women and non-Asian populations [10]. In contrast, there were no significant associations for Asian men. In addition, compared to other ethnic groups, Asians prefer to drink liquor and beer rather than western wine. However, in contrast to beer or spirits, wine consumption appeared to be more beneficial for decreasing the risk of type 2 diabetes [17]. Nevertheless, higher alcohol consumption has also been associated with a greater risk of liver cirrhosis, which is a risk factor for T2D [35]. Taken together, these findings indicate that higher levels of drinking might increase the risk of T2D among Asian men.

The overall RRs of the studies included in this analysis were heterogeneous. The sources of heterogeneity were identified by sensitivity analysis and subgroup analysis. In the sensitivity analysis, we found that no single study significantly skewed the estimates. However, subgroup analysis suggested that higher alcohol intake was associated with age below 50 years old, residence in Japan and Korea, as well as less sample and older studies (before 2010). In addition, the NOS score ≥8 was a source of heterogeneity. The possible reasons for these associations are as follows. First, age is an important risk factor for T2D [36]. Second, the consumption of alcohol in Japan and Korea tends to be higher than in China [37]. Third, there are multiple mediators of the relationship between alcohol intake and T2D, including different risk factors. Nevertheless, the relationship between specific alcohol consumption behaviors and the risk of T2D remains to be elucidated. As the studies included in this systematic review and meta-analysis did not provide sufficient data on the patterns of alcohol consumption, we were unable to account for them in our study, limiting our results to the overall risk rates.

There are several strengths to our meta-analysis. First, we evaluated Asian populations, accounting for regional differences, which has not been done before. Second, we retrieved and pooled results of studies with a prospective design, eliminating selection and recall biases. Third, we performed a dose-response analysis, including a wide range of alcohol doses. Therefore, we evaluated the relationship between drinking and the risk of T2D among men.

Despite these strengths, this study has several limitations. First, the models reported in the evaluated studies were adjusted for a range of variables, which may differentially affect the risk of T2D. Second, the included studies stratified alcohol consumption according to different cutoff values. Third, the self-reported level of alcohol intake during the follow-up period was likely an underestimate. Fourth, we used aggregated data instead of individual-level data, which limits us to more detailed correlation analysis and more comprehensive results.

In conclusion, the results of this study suggest that higher alcohol intake significantly increases the risk of T2D. Furthermore, the dose-response analysis suggests that the association between alcohol consumption and T2D risk follows a J-shaped curve among Asian men, with no reduced T2D risk associated with alcohol intake of 0–57 g/day, and increased risk of T2D associated with alcohol intake >57 g/day. However, further study is needed based on the limitations of the current analysis.

## Figures and Tables

**Table 1:** Characteristics of participants and follow-up in included studies of alcohol consumption in relation to risk of T2D.

Author (year)	Country (follow-up period)	Assessment of exposure	Sample size, *n*	Age at baseline (*y*)	T2D cases, *n*	Categories of highest vs. minimal alcohol consumption	Adjusted factors
Tsumura et al. (1999) [29]	Japan (9.7)	Questionnaires	6362	35-61	456	≥50 g/d vs. never	Age, BMI, regular physical exercise, parental history of diabetes, smoking habits, and FPG level
Lee et al. (2003) [30]	Korea (4)	Self-reported, questionnaires	4055	25–55	83	≥361 g/wk vs. never	Age, BMI, smoking, exercise, family history of diabetes mellitus, and fasting blood glucose
Noriyuki et al. (2003) [31]	Japan (7)	Questionnaires, annual health examinations	2953	35-59	138	≥69.0 g/day vs. never	Age, family history of diabetes, BMI, cigarette smoking, and regular physical activity
Waki et al. (2005) [32]	Japan (10)	Self-administered questionnaire	12913	40–59	703	≥46.1 g/d (men) or ≥11.6 g/d (women) vs. never	Age, BMI, cigarette smoking, exercise, family history of diabetes and prevalent hypertension
Shi et al. (2013) [12]	China (5.4)	Person interviews	51,464	40–74	1,241	≥3 drinks/d vs. never	Age, energy intake, physical activity, smoking, education level, occupation, income level, hypertension, and family history of diabetes.
Lee et al. (2017) [ 13]	Korea (12)	Interview-based questionnaires	1772	40-69	486	≥30 g/d vs. never	Age, BMI, family history of smoking, physical activity, total energy intake and IGI60.
Zhang et al. (2017) [14]	China (4.8)	Questionnaires, health examination	6783	≥45	526	≥20 g/d vs. never	Age, education, smoking factors, central obesity, exercise, family history of diabetes and hypertension
Yatsuya et al. (2018) [15]	Japan (12.2)	Questionnaire survey	3540	35-64	342	>46 g/d vs. never	Age, BMI, smoking status, medication, family history of diabetes, categories of fasting blood glucose and triglycerides

BMI: body mass index; FPG: fasting plasma glucose; FHD: family history of diabetes; METs: metabolic equivalent values; NOS: Newcastle-Ottawa Scale; PA: physical activity; T2D: type 2 diabetes.

**Table 2:** Newcastle-Ottawa Scale (NOS) quality assessment of each cohort study.

Study	Selection				Comparability	Outcome			NOS score
	Representativeness of the exposed cohort	Selection of the nonexposed cohort	Ascertainment of exposure	Demonstration that outcome of interest was not present at start of study	Comparability of cohorts on the basis of the design or analysis	Assessment of outcome	Was follow-up long enough for outcomes to occur	Adequacy of follow-up of cohorts	
Tsumura et al. (1999) [29]	^∗^	^∗^	NA	^∗^	^∗∗^	^∗^	^∗^	^∗^	8
Noriyuki et al. (2003) [30]	^∗^	^∗^	NA	^∗^	^∗∗^	^∗^	^∗^	^∗^	8
Lee et al. (2003) [31]	^∗^	^∗^	NA	^∗^	^∗∗^	^∗^	NA	^∗^	7
Waki et al. (2005) [32]	^∗^	^∗^	NA	^∗^	^∗∗^	^∗^	^∗^	^∗^	8
Shi et al. (2013) [12]	^∗^	^∗^	NA	^∗^	^∗∗^	^∗^	NA	^∗^	7
Lee et al. (2017) [13]	^∗^	^∗^	NA	^∗^	^∗∗^	^∗^	^∗^	^∗^	8
Zhang et al. (2017) [14]	^∗^	^∗^	NA	^∗^	^∗∗^	^∗^	NA	^∗^	7
Yatsuya et al. (2018) [15]	^∗^	^∗^	NA	^∗^	NA	^∗^	^∗^	^∗^	6

^∗^: 1 point, ^∗∗^: 2 points, NA: no point.

## Data Availability

This is a meta-analysis, and all data sources have been presented in the manuscript.

## Additional Points

*Summary Box*. What is already known about this subject? Cohort studies have shown that moderate levels of alcohol consumption may reduce the risk of type 2 diabetes (T2D); however, the findings remain inconclusive. Previously reported reductions in risk among moderate alcohol drinkers might be confined to women and non-Asian populations. Previous meta-analysis examined the shape and strength of the relationship between alcohol consumption and the risk of T2D. However, the impact on Asian populations remains unclear. What are the new findings? This is the first meta-analysis to evaluate the effect of alcohol consumption on the risk of T2D among Asian men. High levels of alcohol consumption are associated with increased risk of T2D among men. Heavy alcohol consumption should be discouraged. How might these findings impact clinical practice in the foreseeable future? Risk assessment for T2D should account for alcohol consumption. Confirming the impact of moderate alcohol consumption on the risk of T2D requires further research. These findings apply to Asian men. Separate research in this area is required for Asian women.

## Supplementary Materials

Supplementary 1 Supplementary Table 1: subgroup analysis of T2D risk for alcohol intake versus the lowest intake.

Supplementary 2 Supplementary Figure 1: the forest plot of alcohol consumption and the risk of T2D (drinking vs. none drinking).

Supplementary 3 Supplementary Figure 2: sensitivity analysisl plot for the association between alcohol intake and the risk of T2D (A. drinking vs. none drinking; B. highest intake vs. lowest).

Supplementary 4 Supplementary Figure 3: funnel plot for the association between alcohol intake and the risk of T2D (A. drinking vs. none drinking; B. highest intake vs. lowest).
