# Supplementary material for: The Dose-Response Relationship between Alcohol Consumption and the Risk of Type 2 Diabetes among Asian Men: A Systematic Review and Meta-Analysis of Prospective Cohort Studies
Source: J Diabetes Res. 2020 Aug 24;2020:1032049. doi: 10.1155/2020/1032049 (PMC7463364; doi:10.1155/2020/1032049)
Supplement: Supplementary 1 — Supplementary Table 1: subgroup analysis of T2D risk for alcohol intake versus the lowest intake. [file 1032049.f1.pdf]

Supplement Table 1 Subgroup analysis of T2D risk for alcohol intake versus the lowest intake.

Subgroup	Alcohol intake vs. none alcohol intake			Highest vs.lowest category		
	Number of database	RR(CI 95%)	P_{Het}	Number of database	RR(CI 95%)	P_{Het}
Age						
＜50 years old	5	0.98(0.82,1.17)	0.003	5	1.19(1.03,1.38)	0.859
≥50 years old	3	1.07(0.81,1.41)	0.001	3	1.15(0.92,1.44)	0.06
Region						
Japan	4	1.03(0.87,1.23)	0.01	4	1.19(1.03,1.38)	0.736
Korea	2	0.98(0.54,1.78)	0.006	2	0.96(0.73,1.27)	1
China	2	0.98(0.73,1.31)	0.009	2	1.32(1.02,1.71)	0.045
Follow-up durations						
＜5 years	2	1.02(0.86,1.21)	＜0.001	2	1.20(1.00,1.44)	0.383
≥5 years	6	0.96(0.60,1.48)	0.021	6	1.15(1.01,1.32)	0.283
Study time						
Before 2010	4	0.93(0.74,1.16)	0.002	4	1.17(1.00,1.36)	0.836
After 2010	4	1.09(0.88,1.35)	0.001	4	1.18(0.98,1.42)	0.097
Sample						
＜10000	6	1.01(0.86,1.19)	0.007	6	1.23(1.08,1.39)	0.874
≥10000	2	1.01(0.72,1.40)	＜0.001	2	1.08(0.81,1.44)	0.059
Cases						
＜500	5	0.98(0.81,1.19)	0.007	5	1.21(1.03,1.41)	0.796
≥500	3	1.05(0.83,1.33)	＜0.001	3	1.14(0.93,1.39)	0.077
NOS score						
＜8	4	0.97(0.79,1.20)	0.002	4	1.14(0.93,1.41)	0.153
≥8	4	1.05(0.86,1.28)	0.005	4	1.20(1.05,1.38)	0.702
