# Supplementary material for: The Dose-Response Relationship between Alcohol Consumption and the Risk of Type 2 Diabetes among Asian Men: A Systematic Review and Meta-Analysis of Prospective Cohort Studies
Source: J Diabetes Res. 2020 Aug 24;2020:1032049. doi: 10.1155/2020/1032049 (PMC7463364; doi:10.1155/2020/1032049)
Supplement: Supplementary 4 — Supplementary Figure 3: funnel plot for the association between alcohol intake and the risk of T2D (A. drinking vs. none drinking; B. highest intake vs. lowest). [file 1032049.f4.pdf]

Supplementary file

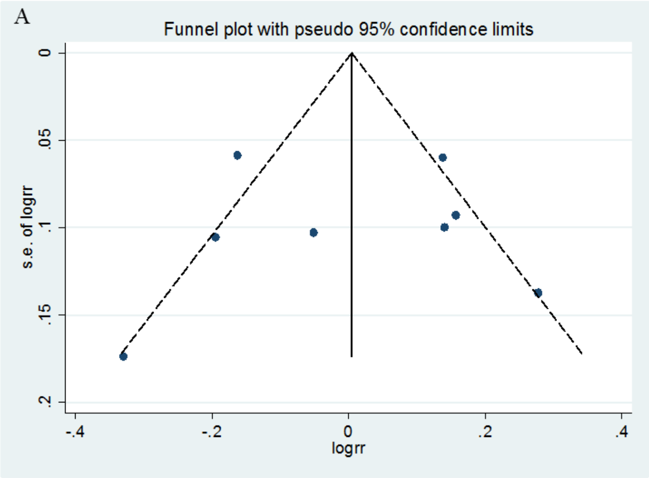

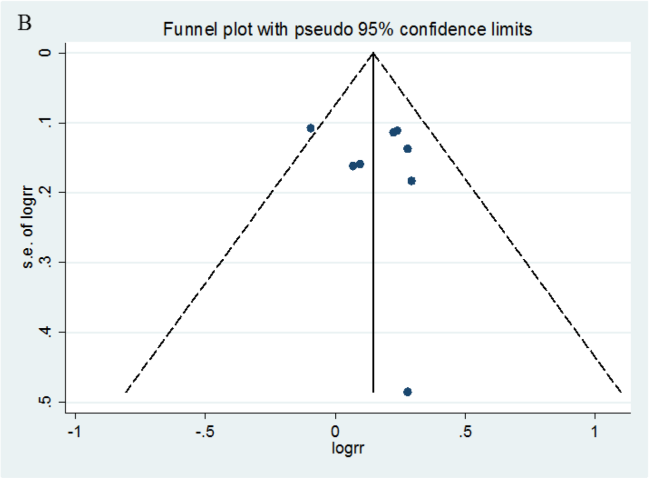

Supplementary Figure 3. Funnel plot for the association between alcohol intake and the risk of T2D (A. drinking vs. none drinking; B. highest intake vs. lowest).
